# Delayed Surgical Intervention in Acute Subdural Hematoma

**DOI:** 10.7759/cureus.11592

**Published:** 2020-11-20

**Authors:** Omar S Akbik, Robert Starling, Ross Green, Yiliang Zhu, Jeremy Lewis

**Affiliations:** 1 Neurosurgery, University of New Mexico, Albuquerque, USA; 2 Statistics, University of New Mexico School of Medicine, Albuquerque, USA

**Keywords:** acute subdural hematoma, delayed surgical intervention

## Abstract

Background

Current guidelines recommend an acute subdural hematoma (ASDH) with a thickness greater than or equal to 10 mm or a midline shift greater than or equal to 5 mm be evacuated regardless of Glasgow Coma Scale (GCS). A large craniotomy versus craniectomy is the preferred surgical treatment for ASDH. A subset of patients who are typically older if not elderly meet the above criteria but have a monitorable neurologic exam. These patients can be followed and taken in a delayed manner allowing the ASDH to become chronic. The delay in treatment allows for a smaller surgery in regards to size of incision, size of craniotomy, and duration of anesthesia.

Methods

Between February 2013 and July 2019, we retrospectively identified 19 patients who underwent delayed evacuation of an ASDH, with the primary outcome being Glasgow Outcome Score (GOS) at discharge and three-month follow-up.

Results

Eight patients (42%) were female and 11 patients (58%) were male. The median age was 77 years, with a range from 49 to 93 years. Sixteen patients (84%) were 60 years of age or older. Mechanism of injury was a fall for 10 patients (53%). Median number of days from initial evaluation and surgical evacuation was 11 days with a range from 6 to 31 days. Thirteen patients (68%) had a GOS of 4-5 at three-month follow-up. Six patients (32%) had a GOS 1-3 at three-month follow-up. Two mortalities (11%) recorded in the postoperative period.

Conclusion

Surgically evacuated ASDH in the elderly population is known to carry a significant mortality and morbidity. With close neuromonitoring, delayed intervention in older patients with an ASDH, initially meeting surgical criteria with a good neurologic exam, is a safe practice. Delayed treatment allows for smaller surgery, decreased operative time, and decreased surgical risk which affects older patients even more than younger patients.

## Introduction

Current guidelines recommend that any acute subdural hematoma (ASDH) with a thickness greater than 10 mm or a midline shift greater than 5 mm on CT scan be surgically evacuated regardless of the patient’s Glasgow Coma Scale (GCS) score [1]. Debate still remains as to the optimal procedure whether a decompressive hemicraniectomy or a craniotomy [2]; however, it is general agreed that a large bony opening should be made in order to properly evacuate acute clot.

Patients 65 years and older undergoing surgical evacuation of an ASDH are statistically correlated with poorer outcomes [3]. Within the elderly population undergoing surgical evacuation of an ASDH, preoperative GCS has consistently been shown to be correlated with outcomes [4]. Longer operative time is associated with greater postoperative complications [5].

However, the authors propose that an older subset of patients who meet the above criteria with an intact or near intact exam may be operated on in a delayed manner. Preoperative GCS has been shown to be a significant factor for postoperative outcome in elderly patients undergoing subdural hematoma (SDH) evacuation [4]. These patients represent the subset of elderly ASDH with the highest probability of doing well postoperatively. Any modifiable factors that can assist with ensuring a better postoperative course should be investigated. A delayed intervention would allow for acute clot to become chronic subsequently allowing for a smaller incision, smaller craniotomy, less blood loss, and duration of anesthesia.

## Materials and methods

Study population and data collection

**Figure 1:**
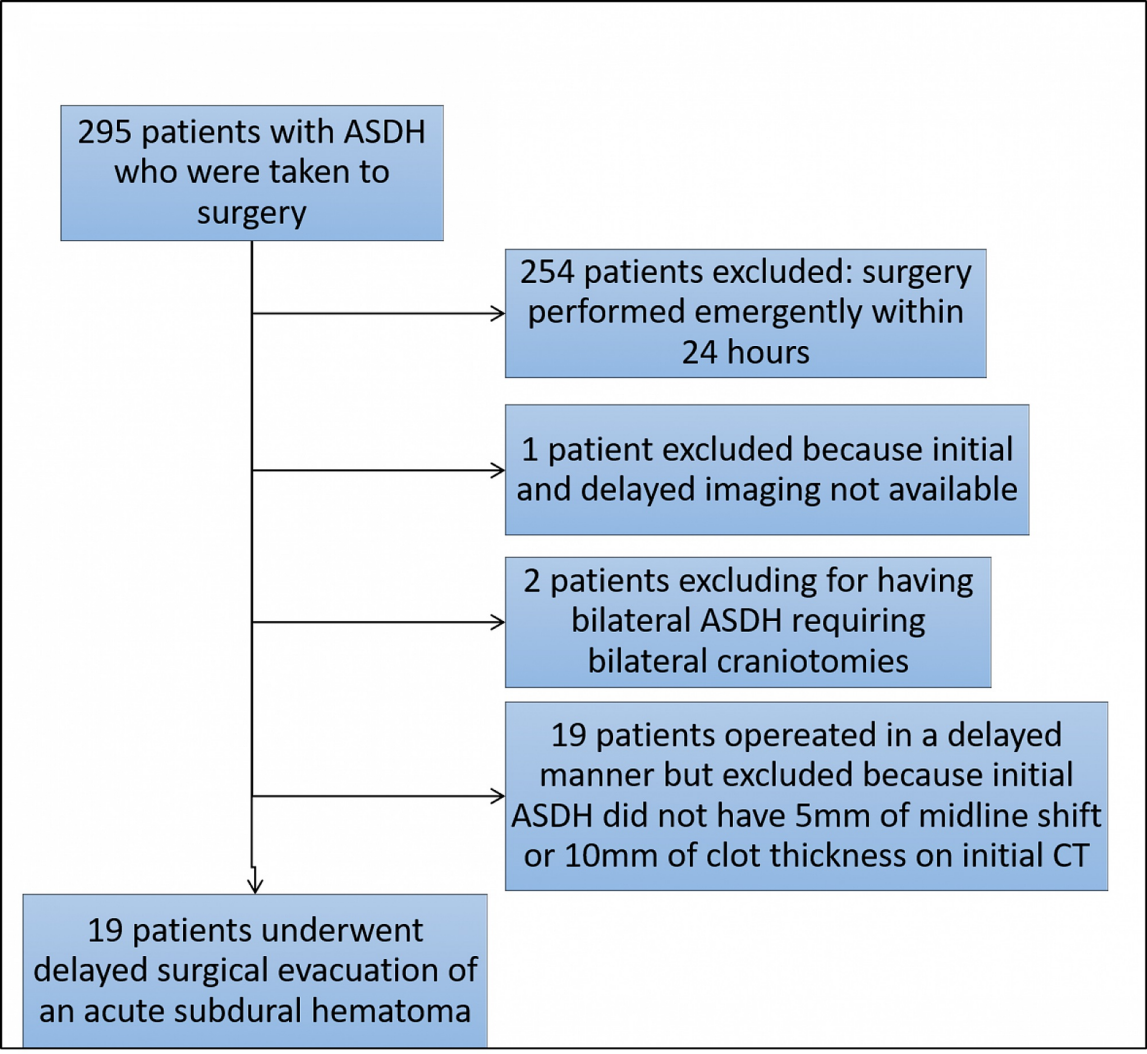
Flowchart of patients included and excluded from analysis ASDH, acute subdural hematoma

We conducted a retrospective case series study identifying all patients with an ASDH with either 5 mm of midline shift or 10 mm of clot thickness who underwent a delayed surgical evacuation at our Level I trauma center (University of New Mexico Hospital) between February 1, 2013 to July 23, 2019. Using International Classification of Diseases, 9th Revision/International Classification of Diseases, 10th Revision and Current Procedural Terminology (CPT) codes, all patients with a diagnosis of ASDH and CPT code for craniotomy for subdural hematoma evacuation were identified. A neurosurgeon reviewed all imaging verifying an ASDH. On review of imaging, the midline shift and clot thickness on initial imaging as well as midline shift and clot thickness on imaging before surgery was recorded. Any patient who was taken emergently (within 24 hours of admission) was excluded. Thirty-nine patients were identified as having delayed intervention for an ASDH. One patient was excluded because both initial imaging identifying the acute subdural and imaging before surgery were not available. Only patients (n = 19) with initial imaging of an acute subdural with either 5mm or more of midline and/or 10mm or more of clot thickness were included in the final analysis (Figure 1).

For the 19 patients meeting all inclusion criteria, hospital records were used to document sex, age, date of initial injury, date of surgery, length of delay between injury and surgery, duration of hospitalization, initial symptoms on first imaging and before surgery including GCS score, preoperative antithrombotic agents, type of surgery performed, duration of surgery, pre- and post-operative Hgb, postoperative GCS, discharge GOS, three-month follow-up GOS, and six-month follow-up GOS.

Criteria for going to surgery, timing to surgery, and type of procedure were at the discretion of the on-call neurosurgery attending. Unless there is a contraindication, the standard practice at our institution is to intervene with surgical evacuation in the face of worsening neurologic exam or worsening symptoms such as headache, altered mental status, etc. Typically, any motor deficit would be a rationale for immediate surgical intervention. In three of the patients, there was a pronator drift that ultimately resolved on repeat examination and a patient who was post ictal who also improved back to baseline. Since the patients returned back to a symmetric examination, surgery was delayed in an attempt to reduce the burden of surgical risk. All antithrombotic agents were reversed at the initial image findings indicating an ASDH. Neurosurgical clinic notes were used to calculate follow up GOS scores.

Statistical analysis

Descriptive statistics were presented to describe the demographics and clinical presentation of the patient sample. We used Fisher’s test to quantify the association between two categorical variables particularly the type of surgery and surgery outcomes; we used the Kruskal test to compare group differences in surgery outcome by surgery type. We also conduct logistic regression analysis to examine GOS at discharge and GOS at three-month follow-up and its relation with demographic and clinical factors.

## Results

Baseline characteristics

**Figure 2:**
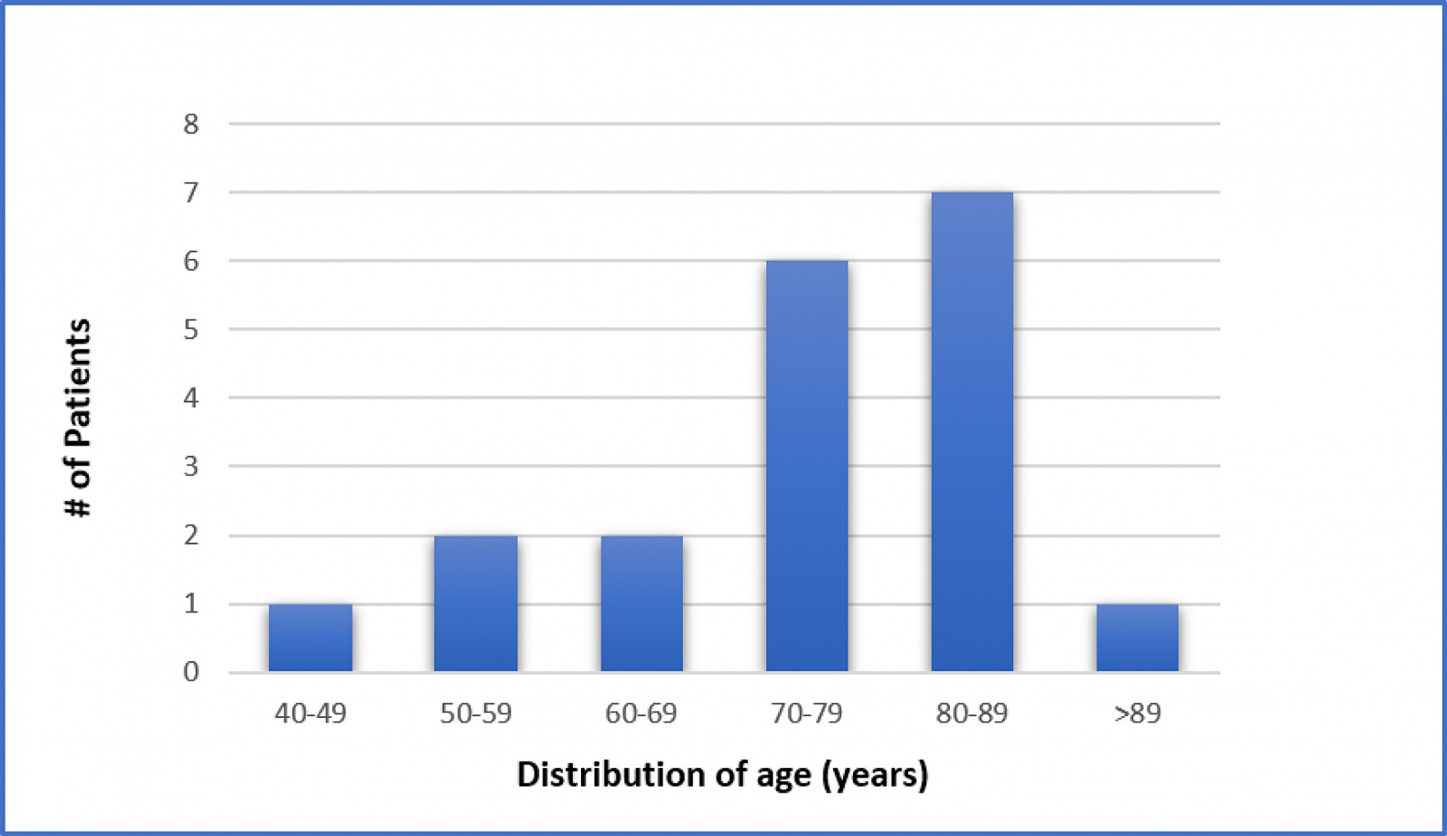
Age distribution in decades

Nineteen patients were included in the final analysis. The median age was 77 years, with a range from 49 to 93 years. Fourteen patients (74%) were 70 years of age or older (Figure 2). Eight patients (42%) were female, and 11 patients (58%) were male. Mechanism of injury was a fall for 10 patients (53%), unknown/found down for five patients (26%), assault for two patients (11%), and motor vehicle collision for two patients (11%). Median number of days from initial evaluation (suspected day of injury) and surgical evacuation was 11 days with a range from six to 31 days (Table 1).

 

**Table 1:** Patient demographics and clinical presentation GCS, Glasgow Coma Scale

Patient Demographics and Clinical Presentation		
Female sex: n (%)	8	42%
Male sex: n (%)	11	58%
Age (year): median (range)	77	49 – 93
Injury to surgery (days): median (range)	11	6 – 31
Injury due to fall: n(%)	10	53%
Midline shift (mm) @1^st^ CT: median (range)	6	0 – 12
Pre-op Midline shift (mm): median (range)	11	6 – 15
Thickness @ 1^st^ CT (mm): median (range)	11	7 – 20
Pre-op thickness (mm): median (range)	16	10 – 23
Initial GCS: median (range)	15	13 – 15
Pre-op GCS: median (range)	14	9 – 15
Lateralizing exam on the day of injury: n (%)	3	16
Lateralizing exam before surgery: n (%)	12	63

Seven patients (37%) had right-sided SDH and 12 patients (63%) had left-sided SDH. Sixteen patients (84%) had a small craniotomy (<4cm) and three patients (16%) had a medium size craniotomy (6-7 cm). Two patients required a second surgery. One was secondary to an acute bleed after a drain was removed. The second patient developed a postoperative epidural hematoma which required evacuation. Eleven patients (58%) had some type of antithrombotic on initial evaluation including ASA, Plavix, and/or Coumadin which treated with platelets, fresh frozen plasma, or 4-factor prothrombin complex concentrate until labs normalized or a stability CT scan was obtained. Patients that were treated in a delayed manner were kept off of their antithrombotic agent during that time.

Radiographic characteristics

All patients had an ASDH on initial CT evaluation. Eleven patients had midline shift of 5mm or more on initial CT head. Sixteen patients had 10mm or more of clot thickness on the initial CT head. On imaging immediately before surgery, all patients had 6mm or more of midline shift and 10mm or more of clot thickness. The median midline shift on initial CT was 6mm which increased to a median value of 11mm on the CT before surgery. The median clot thickness on initial CT was 11mm which increased to a thickness of 16mm on the CT before surgery. There was an 83% increase in the midline shift and a 45% increase in clot thickness from initial CT evaluation after injury to CT evaluation before surgery.

Preoperative characteristics

On initial evaluation on the suspected day of injury, all patients were symmetric in motor exam except three patients (72, 79, 80 years of age) who had asymmetrical motor exams including a pronator drift and post ictal symptoms that improved with initial observation. Presenting symptoms included five patients (26%) with only headaches, four patients (21%) with confusion from baseline cognition per family, one patient (5%) with dizziness, two patients (11%) with a witnessed seizure, one patient (5%) with aphasia, and four patients with no symptoms. On initial evaluation, all patients had a GCS ≥ 13 except one patient who had an initial GCS of 9. That patient was found down and intubated on scene with subsequent extubation after sedation was allowed to wear off. 

Surgery was performed in a delayed manner for all patients. Seven patients (37%) had symmetrical motor exam and 12 patients (63%) had asymmetry on physical exam (within 24 hrs) before surgery. Nine patients (47%) went from a symmetrical to an asymmetrical motor exam. Seven patients (37%) had persistent seizures and/or aphasia. Three patients (16%) had worsening headaches as their main symptom. Thirteen patients (68%) had a GCS ≥ 13.

Operative characteristics

For the patients in this study undergoing delayed surgery, the median duration of surgery was 77 min with a range of 38-125 min (n = 18). The median change in Hgb was 0.9 (g/dL) with a range 0.3-2.1 (g/dL) (Table 2). In order to make any comparative statement on length of surgery and blood loss, we reviewed the surgical times and changes of Hgb of elderly patients undergoing emergent evacuation of an ASDH using larger craniotomies/craniectomies. 

**Table 2:** Intraoperative and postoperative results SDH, subdural hematoma; GOS, Glasgow Outcome Score

Intraoperative and Postoperative Results		
Right SDH: n (%)	12	63%
Left SDH: n (%)	8	37%
Craniotomy size <4cm: n (%)	16	84%
Surgical time (min): median (range)	77	38-125
Drop in Hgb (g/dL): median (range)	0.9	0.3-2.1
Discharge Location: n (%)		
Home	3	16%
Inpatient rehab	11	58%
Skilled nursing facility	3	16%
Morgue	2	11%
Discharge GOS: n (%)		
1	2	11%
2	1	5%
3	8	42%
4	4	21%
5	4	21%

For this emergent surgery cohort (n = 59), the median surgical duration was 102 min with a range of 54-178 min. The difference in operative time between the delayed (smaller craniotomy) and emergent (larger craniotomy/craniectomy) cohorts was highly significant (Wilcoxon test: p-value = 0.001; 95%CI: -37.0, -9.0). For the emergent surgical cohort (n = 52), the median change in Hgb was 2.4 with a range of 0.6-6.4 (g/dL). The difference in the drop in Hgb before and after surgery between the delayed (smaller craniotomy) and emergent (larger craniotomy/craniectomy) cohorts was highly significant (Wilcoxon test: p-value < 0.00001; 95%CI: -2.00, -0.80).

Postoperative outcome

**Figure 3:**
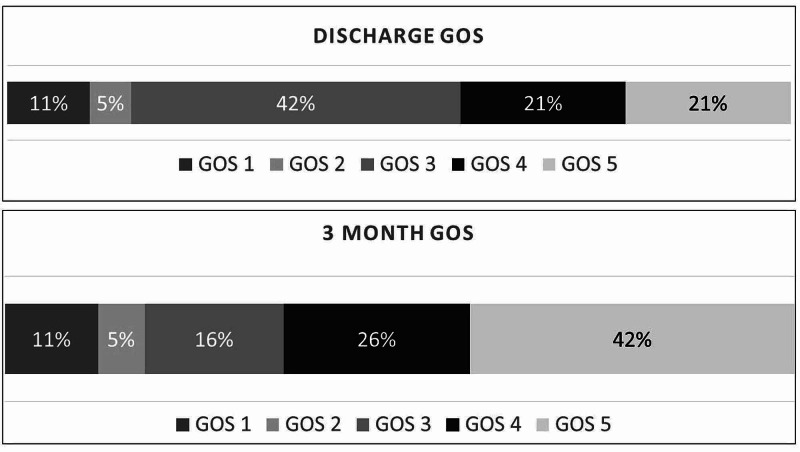
Distribution of GOS at discharge and three-month follow-up GOS, Glasgow Outcome Score

The median duration of hospitalization after surgery was 9 days. Distribution of discharge location include three patients (16%) to home, 11 patients (58%) to inpatient rehab, three patients (16%) to SNF, and two patients (11%) to the morgue. There were two perioperative mortalities (93 and 83 years of age). One patient had a subdural drain pulled after the initial surgery which led to a rebleeding event. Patient had a second surgery but ultimately never recovered and passed away. A second patient had a craniotomy for SDH evacuation, did well neurologically, but developed ischemic colitis and ultimately passed away after going to comfort measures only. Discharge GOS was GOS 5 for four patients (21%), GOS 4 for four patients (21%), GOS 3 for eight patients (42%), GOS 2 for one patient (5%), and GOS 1 for two patients (11%). Three-month follow-up GOS was GOS 5 for eight patients (42%), GOS 4 for five patients (26%), GOS 3 for three patients (16%), GOS 2 for one patient (5%), and GOS 1 for two patients (11%) (Figure 3).

In an analysis of pairwise association, we found post-op GOS 4 and 5 were more likely to be associated with younger patients (median age = 62) as compared with GOS 1-3 (median age = 80) (p = 0.01). Preoperative clot thickness as measured on CT before surgery reached significance with a larger clot thickness associated with a better GOS (4-5) (p = 0.02) (Table 3). At three-month post-op follow-up, however, only patient age remained associated with a GOS (4-5) (p=0.03). However, operative duration trended towards significance from discharge to three-month follow-up analysis (Table 4).

**Table 3:** Discharge GOS analysis GOS, Glasgow Outcome Score

Discharge GOS Analysis			
	GOS 1-3 n=11 (58%)	GOS 4-5 n=8(42%)	p-value
Female Gender: n (%)	5 (62%)	3 (38%)	1.0
Age: median (range)	80(70 – 93)	62(49-85)	0.01
Initial midline shift(mm): median (range)	4 (0 – 10)	7 (2 – 12)	0.17
Pre-op midline shift	11(6 -15)	12.5 (6 – 15)	0.38
Initial thickness: median (range)	10 (7 – 14)	12 (8 – 20)	0.07
Pre-op thickness: median (range)	13(10 – 21)	17 (14 – 23)	0.02
Days delayed: median (range)	12 (6 – 30)	11 (7 – 31)	0.83
Operation duration: median (range)	73 (38 – 100)	93 (48 – 169)	0.11

**Table 4:** Three-month follow up GOS analysis GOS, Glasgow Outcome Score

Three-Month Follow-up GOS Analysis			
	GOS 1-3 n=6(32%)	GOS 4-5 n=13 (68%)	p-value
Female Gender	2(33%)	6 (46%)	1.00
Age: median (range)	84 (70 – 93)	72 (49 – 85)	0.03
Initial midline shift(mm): median (range)	4 (0 - 7)	6.5 (2 – 12)	0.11
Pre-op midline shift	8(6 – 15)	12 (6 - 15)	0.16
Initial thickness: median (range)	11 (10 -14)	11 (7 – 20)	0.79
Pre-op thickness: median (range)	15.5 (12 – 21)	16 (10 - 23)	0.76
Days delayed	13 (10 – 30)	11.5 (7 -31)	0.96
Op duration	68 (38 – 92)	86 (40 – 169)	0.07

## Discussion

ASDH with clot thickness equal or greater than 10mm and/or midline shift equal or greater than 5mm is considered a surgical emergency by standard guidelines [1]. The practice at our institution is to use a larger (>10) craniotomy versus craniectomy for acute subdural clot evacuation. This allows for appropriate exposure and avoids reaching underneath a craniotomy site to evacuate clot which could cause bleeding that is not easily visualized or controllable. 

The American guidelines do not discuss the role of delayed surgery in patients with an ASDH meeting the above criteria without neurologic deficits which is typically an older subdural population. In many instances, we elected to closely monitor these patients with serial neurologic exams and imaging with the knowledge that surgery would be needed in the future. This delayed intervention would allow the acute clot to become subacute to chronic and more amenable to a smaller craniotomy for drainage. The advantage of this approach is less operative time, less anesthesia for an elderly patient as well as a smaller incision including a smaller craniotomy and less trauma to the scalp tissue including the temporalis. While this practice is commonplace in many institutions, there is no literature looking at this specific subset of patients who meet surgical criteria on initial radiographic examination.

Procedure duration has been shown to be associated with increased complications in the elderly population. In the general population, Kim et al. showed that operative duration was correlated with increased complications [6]. Hersey et al. reviewed 4,947 patients 65 and older undergoing lumbar fusion and found that each incremental hour of operative time was associated with increased total postoperative risk including thromboembolism [5].

We compared the average surgical duration of the delayed intervention patients, 77 min, to that of our previously published cohort of elderly patients undergoing emergent evacuation for ASDH, 102 min, and found a statistically significant difference. All patients from that study [4] received large craniotomies or craniectomies for emergent intervention of an ASDH which include a traditional trauma style question mark incision. This is in contrast to delayed intervention patients who had craniotomies typically less than 4cm and a single linear incision above the superior temporal ridge.

Oh et al. showed that EBL and procedure duration are significantly associated with postoperative pneumonia following meningioma resection [7]. Specifically in the elderly population, Asano et al reported that intraoperative blood loss >350mL and a decrease of hemoglobin levels > 2.0 g/dL from preoperatively to postoperatively is significantly associated with increased risk for postoperative systemic complications [8]. In this study, the average drop in Hgb for delayed intervention patients was 0.9g/dL. Again, comparing changes in Hgb to our previously studied cohort of elderly ASDH patients undergoing larger craniotomies, we found that the average change in Hgb was 2.4g/dL and that a majority of those patients were transfused with products. 

While the patient population of the current study, delayed intervention, and the patient population of the prior study, emergent evacuation, display two different patient populations and baseline characteristics, the authors felt that it would be of value to review the surgical duration and change in Hgb of both patient populations in an attempt to better quantify a small craniotomy vs a large craniotomy. 

It is important to reiterate, that the authors would never advocate delaying surgery for elderly patients with an ASDH causing mass effect/midline shift with neurologic deterioration. Any theoretical decrease in risk from a shorter surgery would be negligible next to a permanent deficit or even herniation. However, in an elderly patient with an ASDH with no lateralizing signs and near-normal GCS who meets criteria for surgery (≥5mm of midline shift and/or ≥10mm of clot thickness), a rationale can be made for observation in an attempts to decrease the surgical intervention and accompanied surgical risk associated with a bigger procedure. The above is also contingent on having a center where close neurologic observation can be performed.

In regards to outcome, 68% of the patients in this study had a good outcome (GOS 4-5) at three-month follow-up. Age was the only statistically significant factor with outcome at three-month follow-up. The mortality rate was 11% at discharge and both of those mortalities were secondary to complications of surgery (ischemic colitis and rebleed after drain removal). At three months, 32% of patients had a poor outcome (GOS 1-3). When comparing our results of the emergent intervention for ASDH to the delayed intervention for ASDH cohort, at three-month follow-up, 27% of patients undergoing emergent surgery had a GOS 4-5 as compared to 68% for delayed intervention. The discharge mortality rate was 39% as compared to 11% for the emergent vs delayed intervention cohort. It is imperative to note that these two cohorts represent different patient populations who are expected to have different outcomes. However, the data at least shows what we expected which is a significantly lower mortality rate and a higher percentage of good outcomes at three-month follow-up. 

Choi et al. published a series of 18 patients who had delayed evacuation of an ASDH reporting good outcomes in 89% of their patients with a discharge GOS 4-5 [9]. Their inclusion criteria were any ASDH that later underwent burrhole evacuation, not necessarily those that met surgical criteria by current guidelines. Of their 18 patients, 14 patients did meet surgical criteria with an ASDH with 10 mm of clot thickness and/or 5 mm of midline shift. Their average age was 67 years (range 38 to 83) with a mean delayed operation day of 13.9 days (range 7-28) similar to our cohort of 19 patients with an average age of 77 years (range 49 -93) and mean delayed operation day of 11 days (range 6-31). Choi et al. reported excellent outcomes with a discharge GOS of 4-5 in 89% of their patients. We reported a GOS of 4-5 at three-month follow-up in 68% of patients which may be due to the older age of our cohort versus theirs.

While numerous publications have looked at the risk factors for any ASDH ultimately requiring surgical intervention after initial conservative management [10,11], those publications typically look at patients with an ASDH that is less than 1 cm thick or with 5 mm of midline shift. However, this study reports on a subset of patients who meet surgical criteria at the onset but have delayed surgical intervention in the aims of reducing the size of surgery and duration as well as associated risks.

In Germany, Petridis et al. published a decision tree for the management of acute subdural in the elderly population, greater than 65 years of age [12]. Patients that presented with a GCS of 13-15 (n = 18) were observed and taken if they decompensated. The mortality rate for that subgroup in their review was 22% at discharge which is in line with our results of 11% at discharge. While they found preoperative midline shift, not subdural thickness, to be associated with GOS at discharge. We found preoperative thickness, not midline shift, to be associated with GOS at discharge. They did not have to follow up data on their cohort, but our three-month follow-up analysis showed that age was the only significant factor associated with three-month GOS with operative duration trending towards significance. 

While the current American guidelines do not make a distinction between the management of acute subdural based upon age, we have found that there exists a subpopulation of patients, typically > 60 years of age, with acute subdural hematoma who can be watched although they have more than 5mm of midline shift or clot thickness greater than 10 mm. They can be taken in a delayed manner with a good prognosis. 

There are inherent limitations in this practice in that a center must be equipped to be able to closely monitor these patients for worsening neurologic exam which would necessitate a more urgent surgical procedure.

## Conclusions

Current guidelines recommend that all ASDH with 10 mm of clot thickness and/or 5 mm of midline shift be evacuated immediately regardless of GCS. There does exist a patient population, typically >60 years of age, who meet the above surgical criteria and have a good neurologic exam (GCS 13-15). This subset of patients may benefit from a delayed surgery allowing for the acute blood to become more chronic allowing for a smaller craniotomy with less operative blood loss and less operative time, exposing the patient to less surgical risk. 

## References

[1] Bullock MR, Chesnut R, Ghajar J (2006). Surgical management of acute subdural hematomas. Neurosurgery.

[2] Phan K, Moore JM, Griessenauer C (2017). Craniotomy versus decompressive craniectomy for acute subdural hematoma: systematic review and meta-analysis. World Neurosurg.

[3] Wilberger JE, Jr. Jr., Harris M, Diamond DL (1991). Acute subdural hematoma: morbidity, mortality, and operative timing. J Neurosurg.

[4] Akbik OS, Starling RV, Gahramanov S, Zhu Y, Lewis J (2019). Mortality and functional outcome in surgically evacuated acute subdural hematoma in elderly patients. World Neurosurg.

[5] Hersey AE, Durand WM, Eltorai AEM, DePasse JM, Daniels AH (2019). Longer operative time in elderly patients undergoing posterior lumbar fusion Is independently associated with increased complication eate. Global Spine J.

[6] Kim BD, Hsu WK, De Oliveira GS, Jr. Jr., Saha S, Kim JY (2014). Operative duration as an independent risk factor for postoperative complications in single-level lumbar fusion: an analysis of 4588 surgical cases. Spine.

[7] Oh T, Safaee M, Sun MZ (2014). Surgical risk factors for post-operative pneumonia following meningioma resection. Clin Neurol Neurosurg.

[8] Asano K, Nakano T, Takeda T, Ohkuma H (2009). Risk factors for postoperative systemic complications in elderly patients with brain tumors. Clinical article. J Neurosurg.

[9] Choi YH, Han SR, Lee CH, Choi CY, Sohn MJ, Lee CH (2017). Delayed burr hole surgery in patients with acute subdural hematoma: clinical analysis. J Korean Neurosurg Soc.

[10] Laviv Y, Rappaport ZhH (2014). Risk factors for development of significant chronic subdural hematoma following conservative treatment of acute subdural Hemorrhage. Br J Neurosurg.

[11] Mathew P, Oluoch-Olunya DL, Condon BR, Bullock R (1993). Acute subdural haematoma in the conscious patient: outcome with initial non-operative management. Acta Neurochir.

[12] Petridis AK, Dorner L, Doukas A, Eifrig S, Barth H, Mehdorn M (2009). Acute subdural hematoma in the elderly; clinical and CT factors influencing the surgical treatment decision. Cent Eur Neurosurg.

